# Hospital Expenditure.—I

**Published:** 1902-03-01

**Authors:** 


### The Institutional Workshop.

### HOSPITAL EXPENDITURE.—I.

By the Author of "The Hospital Commissariat."

The discussion relating to the cost of alcohol in London hospitals which has lately appeared in these columns raises a very interesting point in connection with the appearance of the recent edition of "Burdett's Hospital Annual" for 1901.

Prior to the first appearance of this Annual in 1889 no systematic attempt had ever been made to compare the rate of expenditure in British hospitals, and the earlier attempts at calculation were almost defeated by the absence of any common system of accounts on which comparison could be based. The uniform system of accounts is now in operation at all important institutions, and there is no longer any ambiguity as to the amount spent under its various headings. It is impossible to over-estimate the value of this admirable system of accounts to the hospitals. But it is unfortunately too true that in rendering it possible to compare the cost of the various items at different institutions it has opened up a hunting-ground for the outside critic full of very dangerous pitfalls.

If the astonishing variety of administration presented by these great institutions be once fairly understood all the variations in expenditure which are revealed by a comparison of their accounts are seen to be perfectly normal. The total cost of each patient per day in many hospitals differing in size, in character, in situation and in administration, is revealed as identical, while the amount spent per day on such separate items as provisions, salaries, domestic expenses, etc., differs to a remarkable extent.

For instance, the cost per patient per day, including the whole expenditure of the hospital, at the following eight hospitals differs by only a few pence, the extreme variation between first and last being only 1s. 2d.

*Total Cost of each Patient per Day.*

| | s. | d. |
|---|---|---|
| The Royal Free Hospital... ... ... ... | 3 | 11 |
| King's College Hospital ... ... ... | 4 | 5 |
| The London Hospital ... ... ... ... | 4 | 6 |
| Guy's Hospital ... ... ... ... ... | 4 | 6 |
| Charing Cross Hospital ... ... ... | 4 | 9 |
| The Middlesex Hospital ... ... ... | 5 | 0 |
| St. Mary's Hospital ... ... ... ... | 5 | 0 |
| University College Hospital ... ... | 5 | 1 |

Yet if we attempt to analyse their accounts separately we are posed by bewildering inconsistencies.

For example, how does University College Hospital manage to victual its household on an average of £18 a year per occupied bed, while the Royal Free Hospital which spends 1s. 2d. a day less on each patient, or £13 a year less on each occupied bed, expends £28 a year, or £10 more for each bed, on the item of provisions?

Again, Guy's Hospital and the London are worked at an identical cost. But examine the details and you will see that Guy's spends £13 more per occupied bed than the London on establishment and miscellaneous charges, while the London spends £10 per occupied bed more than Guy's on salaries.

Nothing could be more unreasonable, though nothing perhaps is more natural, than to argue hastily that as one hospital can show an expenditure of £18 a year on provisions, no other hospital is justified in expending more. And nothing but careful and individual inquiry would satisfy the critic that here is no startling revelation of wholesale extravagance, or reveal to him that the board of all the nurses at University College Hospital is included under the head of salaries, and stands for quite £8 or £10 per occupied bed.

The public do not sufficiently understand the complicated character of the hospital household. They are apt to consider that when the cost of administration and the cost of salaries is deducted, the rest of the money is all spent on the patients.

But in estimating the cost of provisions the patient's cost is only one out of several distinct strands; it stands for little more than half, and it is quite reasonable that it should be so. The cost of the patient's board varies in London hospitals from £13 to £16 a year per bed, or between 5s. and 6s. 6d. a week. The patient remains from three weeks to a month in the wards; part of the time he is probably on low diet, getting nothing but milk or beef tea, and even as a partial convalescent he is entirely quiescent and requires only very simple food.

On the other the medical officers, nurses, and permanent staff give the best years of their life to the institution; their duties are exceptionally arduous and engrossing, and unless their health is to suffer they must be well fed. The cost of boarding the nurses in London hospitals may be reckoned as a yearly charge on each occupied bed of from £6 to £8. This is reckoning an average of three beds to each nurse, and the cost of the nurse's board at from 7s. to 10s. a week.

In addition to the board of the nurses there is the board of the servants, which may be reckoned at an average of another pound or two per occupied bed.

In the matter of medical officers and staff there is a great variation. At some hospitals none of the medical staff receive any board at all. At others, 9, 10, 15 or 18 resident officers receive full or partial board. In the total expenditure these variations are not revealed. What is not expended on board is given in the form of salary, and the result is made the same. But in reckoning up what is spent on food every separate department of the hospital must be allowed its full value. And in hospitals where a large number of the medical staff officers receive board it will be found that an additional sum of from £2 to £4 per occupied bed will be expended on provisions.

Briefly summarised the figures for yearly cost of provisions per occupied bed work out as follows:—

| | | | |
|---|---|---|---|
| Cost of patients ... ... ... | £13 | to | £16 |
| Cost of nurses ... ... ... | 6 | | 8 |
| Cost of servants ... ... ... | 1 | | 2 |
| Cost of medical and other officers | 2 | | 4 |
| Total cost of provisions ... | £22 | to | £30 |

Referring to "Burdett's Annual" for 1901 it will be found that all the hospitals, excepting Westminster, which was partially closed for six months, and University College, in which the board of nurses is reckoned under the head of salaries, fall between these two totals. It is a remarkable testimony to the careful and economical administration of the metropolitan hospitals. For the variations can readily be accounted for by anyone who possesses a knowledge of the elements which go to make up each household.

We should like to see some attempt made in the reports issued by the various hospitals to place subscribers in a position to judge of these things for themselves. We believe that it would be in all respects more satisfactory to make it quite clear that a large resident staff of "persons other than patients" must from the necessities of the case be maintained, and to issue a yearly return stating the number and office of all who live within its walls.

If this were done it would be possible for each hospital to show the cost of boarding their inmates and satisfy not only those who take the trouble to inquire, but the public in general, that the administration is systematic and economical.

But this would still only render it possible to compare the cost *en gros*. So soon as an attempt is made to descend from generalities to particulars and criticise the cost of some special drug, or article of food, or domestic appliance, a new crop of difficulties at once arises, and to these we may be permitted to call attention in a further article.

